# Autocrine ligands of the epithelial growth factor receptor mediate inflammatory responses to diesel exhaust particles

**DOI:** 10.1186/1465-9921-15-22

**Published:** 2014-02-20

**Authors:** Sam Parnia, Lynnsey M Hamilton, Sarah M Puddicombe, Stephen T Holgate, Anthony J Frew, Donna E Davies

**Affiliations:** 1 The Brooke Laboratories, Division of Infection, Inflammation and Repair, School of Medicine, University of Southampton, Southampton General Hospital, Southampton SO16 6YD, UK; 2 Allergy & Inflammation Research, The Brooke Laboratory, Level F South Block, Mailpoint 888, Southampton General Hospital, Southampton SO16 6YD, UK

**Keywords:** Air pollution, Neutrophilia, Inflammation, Epidermal growth factor receptor, Interleukin-8, Transactivation, Ligand shedding

## Abstract

**Background:**

Diesel exhaust is associated with cardiovascular and respiratory mortality and morbidity. Acute exposure leads to increased IL-8 expression and airway neutrophilia, however the mechanism of this response is unknown. Objectives: As cigarette smoke-induced IL-8 expression by epithelial cells involves transactivation of the epidermal growth factor receptor (EGFR), we studied the effects of diesel exhaust particles (DEP) on IL-8 release and the role of the EGFR.

**Methods:**

Primary bronchial epithelial cells (PBEC) were exposed to DEPs or carbon black. IL-8 and EGFR ligand expression (transforming growth factor alpha (TGFα), heparin-binding EGF-like growth factor, and amphiregulin (AR)) were assessed by quantitative RT-PCR and ELISA.

**Results:**

DEP, but not carbon black, caused a dose-dependent increase in mitogen-activated protein kinase (MAPK) activation and IL-8 expression, however above 50 μg/ml there was an increase in cytotoxicity. At 50 μg/ml, DEPs stimulated transcription and release of IL-8 and EGFR ligands. IL-8 release was blocked by EGFR neutralizing antibodies, an EGFR-selective tyrosine kinase inhibitor and by the metalloprotease inhibitor, GM6001, which blocks EGFR ligand shedding. Neutralizing antibodies to AR, TGFα and heparin-binding (HB)-EGF reduced DEP-induced IL-8 by >50%. Conclusion Expression of IL-8 in response to DEPs is dependent on EGFR activation and that autocrine production of EGFR ligands makes a substantial contribution to this response. *Capsule Summary:* This study identifies a mechanism whereby diesel particles stimulates IL-8 release from bronchial epithelial cells. This mechanism may help to explain the recruitment of neutrophils into the airways of people exposed to particulate air pollution.

## Introduction

Diesel exhaust particles (DEPs) are now one of the major contributors to inhalable particulate matter pollution throughout the industrialized world. Although several epidemiological studies have reported associations between daily concentrations of ambient particulate matter (PM) and increased incidences of allergies, asthma, respiratory infections, increased hospitalization for respiratory diseases, decreased pulmonary function, and premature mortality among the general population [1,2], the mechanisms leading to disease have not been fully elucidated.

The mechanism by which DEPs induce their effects is complex and relates to both the physical and chemical properties of the particles. The chemical properties of the particles involved in inflammatory responses are thought to be due to a combination of oxidative stress and the effects arising from the organic fraction of the particles. This is especially complex, as DEPs contain hundreds (or even thousands) of compounds including poly aromatic hydrocarbons, which are known human carcinogens [3].

In studies using both murine models [4] and human volunteers [5], exposure to DEPs causes airway neutrophilia. *In vitro* studies have demonstrated that exposure to DEPs leads to an inflammatory response as demonstrated by an increase in the release of Interleukin-8 (IL-8, CXCL8), Monocyte Chemotactic Protein 1 (MCP1, CCL2) and Regulated Upon Activation, Normally T-Expressed, And Presumably Secreted (RANTES, CCL5) as well as soluble Intercellular Adhesion Molecule 1 (ICAM-1) [6-8]. DEPs activate the p38 mitogen-activated protein (MAP) kinase pathway which leads to the production of IL-8 and RANTES by human bronchial epithelial cells (HBEC) [9]. IL-8 is a potent neutrophil chemoattractant and is produced by bronchial epithelial cells in response to oxidants via activation of nuclear factor kappa B (NFκB) [10]. Many inhaled substances such as cigarette smoke extract (CSE) and DEPs contain oxidants that may play an important role in the recruitment and activation of neutrophils *in vivo*. However studies have demonstrated that the release of this inflammatory mediator in response to CSE also involves other mechanisms, including activation of the Epidermal Growth Factor Receptor (EGFR) [11].

The EGFR is one of the major receptors expressed by epithelial cells and its expression is increased both in asthma [12] and in chronic smokers [13]. Bronchial epithelial cells also produce ligands for the EGFR, which include transforming growth factor α (TGFα) heparin-binding EGF-like growth factor (HB-EGF) and amphiregulin (AR) [14]. These growth factors are produced as transmembrane precursor molecules whose processing and release (ectodomain shedding) is a highly regulated process involving metalloproteinases [15], and functionally contribute to epithelial maintenance and repair. HB-EGF shedding has been associated with transactivation of the EGFR by G-protein coupled receptors [16]. EGF is known to stimulate IL-8 production by primary bronchial epithelial cells [17] and in studies using CSE, we have demonstrated that secretion of IL-8 is partially dependent on EGFR activation via autocrine ligand shedding [11]. As we have demonstrated that *in vivo* exposure to DEPs causes EGFR activation [18] and induction of IL-8 in the bronchial epithelium [19], we investigated the involvement of autocrine ligands in the release of IL-8 from bronchial epithelial cells in response to DEP. Here we show that DEPs is able to induce expression and release of EGFR ligands and that synthesis and release of IL-8 in response to DEP is dependent on EGFR activation by these ligands.

## Materials and methods

Carbon Black (CB) (PM 2.5) was donated by Dr Kelly Berube (University of Cardiff). These particles had been originally obtained from Monarch 880CB (Cabot, Billerica, MA). DEP was purchased from the NIST (National Institute of Standards & Technology, Gaithersburg, MD, USA). DEP stocks were prepared as suspensions in Ultraculture serum free medium (BioWhittaker, Wokingham, UK) at a concentration of 1mg/ml; the suspension was vortexed for 2 minutes before being placed in a sonic water bath for 3 minutes. CB stocks were also prepared in the same manner. DEP was used in the following doses (10, 50, 100, 200 μg/ml). CB was used at 200 μg/ml) The neutralizing polyclonal sheep anti-EGFR antibody was raised against EGF affinity-purified receptors derived from A431 squamous carcinoma cell membranes [20] and was partially purified by (NH_4_)_2_SO_4_ precipitation and diethylaminoethyl (DE-52; Whatman, Maidstone, Kent, UK) ion exchange chromatography [11]. The EGFR-selective tyrosine kinase inhibitor, AG1478 (Biomol Research Laboratories Inc., Plymouth Meeting, PA) and the broad metalloproteinase inhibitor, GM6001 (Chemicon, International, Temecula, CA 92590) were prepared as stock solutions in (dimethyl sulphoxide) DMSO and diluted in medium for use; vehicle controls were performed. Neutralizing antibodies to Amphiregulin, HB-EGF and TGFα were purchased from R & D Systems (Abingdon, Oxford, UK). The doses used were based on our prior experience with bronchial epithelial cells cultures [11]. All measurements were carried out at 6 hour intervals up to 24 hours for all experiments described below aside from the western blot which was carried out at 10, 30 and 60 minute intervals.

### Fiberoptic bronchoscopy and primary bronchial epithelial cell cultures

Bronchial epithelial brushings were obtained by fibreoptic bronchoscopy from non-smoking adult volunteers (N = 10, five males, five females), mean age 34.7 years (range 22–54) and mean FEV_1_ 105.7% (range 95–120%) predicted. All subjects were free from respiratory tract infections for a minimum of 4 weeks before the study. Written informed consent was obtained from all volunteers and ethical approval was obtained from the Joint Ethics Committee of Southampton University and General Hospital. Bronchoscopy was performed using a fiberoptic bronchoscope (FB-20D; Olympus, Tokyo, Japan) in accordance with standard published guidelines [21]. Epithelial cells were obtained using a standard sterile single-sheathed nylon cytology brush. The cells were cultured in Bronchial Epithelial Growth Medium (BEGM) (Clonetics, San Diego, CA) in flasks coated with collagen using Vitrogen-100 (Nutacon, Leimuiden, The Netherlands), as previously described [22]. The epithelial cells were grown as monolayer cultures in BEGM, seeded into 24-well culture trays and used for assays at passage two.

The cells were exposed to BEGM ± DEPs in the presence or absence of 1 μM AG1478, 1 μM GM6001, (a broad MMP Inhibitor), 500 μg/ml sheep anti-EGFR antibody, neutralizing antibody to HB-EGF (4 μg/ml), AR (10 μg/ml) or TGF-α (0.3 μg/ml) for 24 h at 37°C, 5% CO_2_. The conditioned medium was then removed from the cells, clarified by centrifugation, and stored at -80°C until assay. The cells were taken for RNA extraction (see below).

### Lactate dehydrogenase (LDH) assay

The proliferation of primary bronchial epithelial cells (PBECs) was measured as cell number which was determined using the methylene blue uptake assay [23] where A_630nm_ was shown to be proportional to cell number. Cytotoxicity of DEP towards PBECs was determined by monitoring the release of LDH into the cell culture medium. An LDH activity assay kit (Sigma Aldrich, St Louis, USA) was used, which utilizes a colorimetric method for the detection of LDH activity and its ability to catalyse the following reversible reaction: Pyruvic Acid + NADH ←----→ Lactic Acid + NAD. The reaction equilibrium strongly favours reduction of pyruvate to lactate at a rate proportional to the amount of LDH. Lactic acid, NAD and NADH do not absorb significantly in this range. Therefore it is possible to accurately measure changes resulting from the conversion of pyruvic acid to lactic acid due to LDH activity. A calibration curve for serum LDH using known amounts of pyruvate, (corresponding to a specific amounts of LDH activity) was constructed. One vial of NADH was made up in 1 ml of sodium pyruvate, which gives 0.75 mM sodium pyruvate and 1.28 μM NADH. This solution was used immediately due to its unstable nature. The assay was performed by placing 100 μl of substrate solution into 1 ml disposable cuvettes. 10 μl of assay sample (cell supernatant) was added to each cuvette and the solution incubated at 37°C for 30 minutes. After this time, 100 μl of colour reagent was added and the cuvettes were incubated at room temperature for 20 minutes. The reaction was stopped with 1 ml 0.4 M NaOH (0.8 g in 50 mls distilled water). Absorbance was read at 450 nm using a spectrophotometer.

### The methylene blue assay

The methylene blue assay is a colorimetric method that allows estimation of the number of adherent cells present in a microculture and relies on the fact that Methylene Blue is a basic dye that is positively charged at pH 8.5 and binds electrostatically to negatively charged groups within cells (predominately phosphate moieties of nucleic acids and some charged groups in proteins). The medium was removed from the cells which were then fixed with 500 μl/well/10% Formol Saline (9 g NaCl in 100 ml 40% Formaldehyde and 900 ml of Water) for at least 60 minutes. After fixation the plates were washed in running water and then blotted dry. The wells were then stained with 250 μl of 1% w/v Methylene Blue in 0.01 M borate buffer (3.82 g Disodiumtetraborate together with 1% Distilled water) for 30 minutes and then washed with 0.01 M Borate Buffer or running water until no more blue dye was detected in the washing solution, and were then blot dried. The Methylene Blue in the wells was then eluted using 200 μl/well 1:1 (Volume/Volume) ETOH and 0.1 M HCL for each of the wells in the 24 well plate. The eluted methylene blue solution was diluted (1:20) in 1:1 (V/V) ETOH and 0.1 M HCL and made into a final volume of 100 μl in 24 wells of a 96 well plate before being read at A630 using an ELISA plate reader.

### Analysis of gene expression

RNA was extracted using TRIZOL® reagent (Invitrogen, Paisley, UK) according to the manufacturer’s protocol. Total RNA was DNase treated and reverse transcribed using random hexamer primers and AMV reverse transcriptase (RT) (Promega, southampton, UK). The target primer and the probe sequences were as follows:

AR: (forward) gtggtgctgtcgctcttgatac, (reverse) gcttcccagagtaggtgtcattg, (probe) tccaatccagcagcataatggcctga;

HB-EGF (forward) gatctggaccttttgagagtcactt, (reverse) tcccgtgctcctccttgtt, (probe) agccacaagcactggccacacca;

TGF- (forward) ctagttggttctgggctttgatct, (reverse) tggttttgggcatttgagtca, (probe) ttccaacctgcccagtcacagaagg;

IL-8 (forward) aaggaaccatctcactgtgtgtaaac, (reverse) ttagcactccttggcaaaactg, (probe) ctgccaagagagccacggccag

Each sample was assayed in duplicate using 18S rRNA (primers and probe from Applied Biosystems) for normalization. Relative gene expression was determined using the ΔΔCt Method.

#### Western blotting

PBECs were exposed to DEPs for the times specified and cell lysates prepared for western blot analysis. Samples were subjected to SDS PAGE and Western blotting for activated MAPK using a pan MAPK for normalization, as previously described [12].

### Analysis of growth factor and cytokine release

Enzyme-linked immunosorbent assay (ELISA) kits for TGF-α (CN Biosciences, Nottingham, UK.), AR (R&D Systems, Abingdon, UK), and IL-8 (Biosource International, Camarillo, CA) were used according to the manufacturer’s instructions; the minimum detectable amount of each factor in these assays was 10, 15, and 15 pg/ml, respectively.

### Statistical analysis

Continuous data were expressed as the median and interquartile range (IQR). Further analysis was carried out using a Kruskal Wallis test followed by a Wilcoxon Signed Ranked test using SPSS software (SPSS Inc., Chicago, IL). A *p* value of <0.05 was considered to be statistically significant.

## Results

### IL-8 gene expression and release

**Figure 1:**
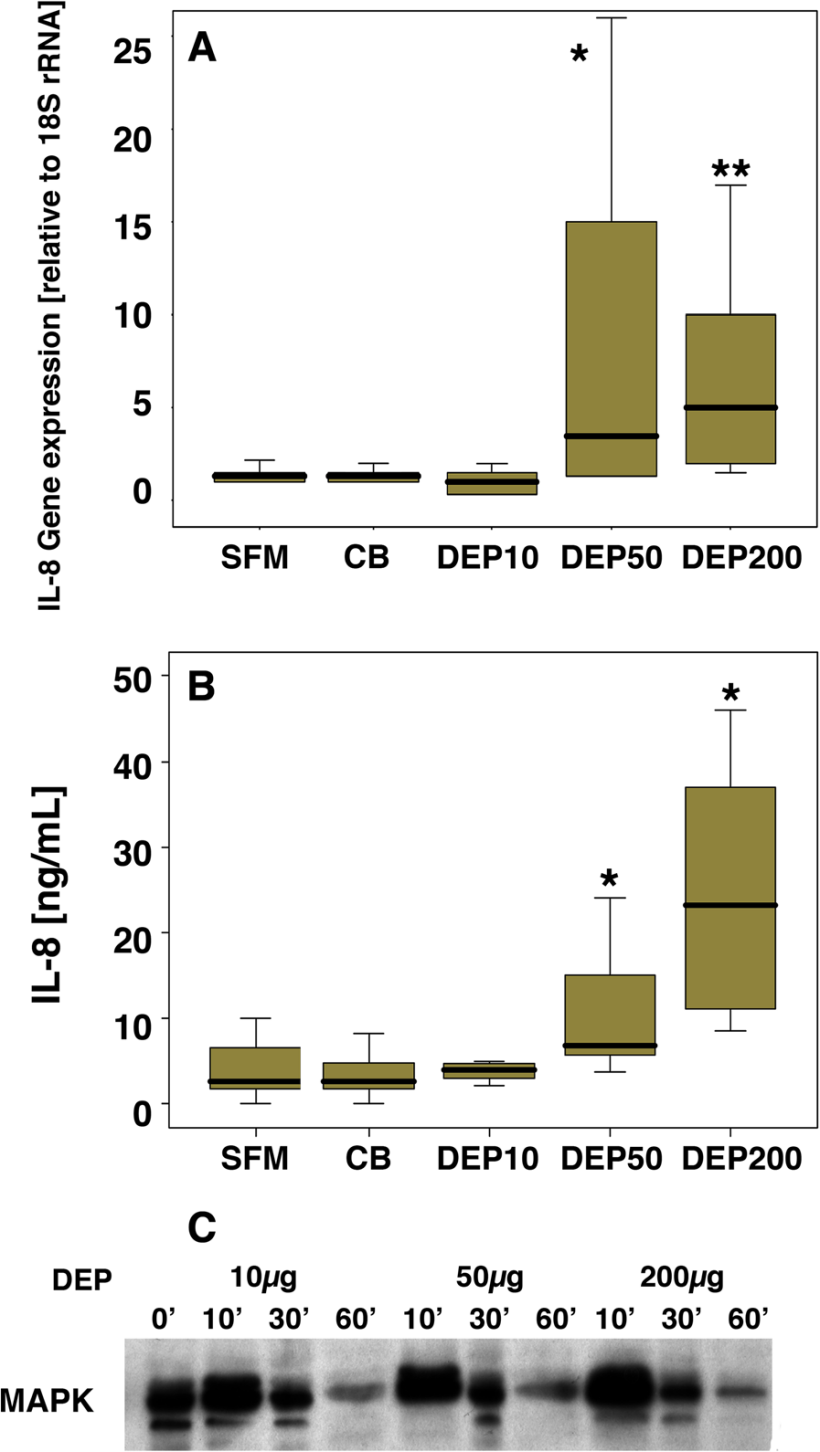
**The effect of DEP on IL-8 mRNA expression (A) and IL-8 protein release (B) by PBECs exposed to DEP in SFM for 18 h and 24 h respectively.** Samples were compared with PBECs cultured in SFM alone. Statistical analysis was carried out using Kruskal Wallis followed by Wilcoxon signed rank test; * p < 0.01 ** p < 0.005 (n = 10). **(C)** a typical western blot obtained after analysis of activation of the MAPK pathway in PBEC samples exposed to 10, 50 or 200 μg/ml DEP for the times indicated. Data are representative of 4 individual experiments.

After 18 h exposure to DEPs at 50 or 200 μg/ml (DEP_50_ or DEP_200_) there was significant induction of IL-8 gene expression in PBECs (Figure 1A). This response was equivalent to 80 ±10% of IL-8 gene expression obtained with TNF-α (10ng/ml) (data not shown). IL-8 protein release at 24 h was also significantly enhanced in response to DEP exposure and was equivalent to 64 ± 5 % of IL-8 release obtained with TNFα (10 ng/ml). Carbon black (CB) (200 μg/ml) did not induce IL-8 gene expression (relative gene expression (median (IQR)): SFM = 1.3 (0.5-2.0) *versus* CB 200 μg/ml = 1.5 (0.8-1.6) (p > 0.05), nor did it affect basal IL-8 release (IL-8 ng/ml (median (IQR)): SFM = 2.2 (1.0-7.0) *versus* CB = 2.0 (1.0-3.2) p > 0.05), suggesting that the inflammatory effects of DEPs as measured by the release of IL-8, are due to the adsorbed substances on the surface of the particles, rather than the carbon core. Although DEPs can mediate some of their effects via oxidative stress [24], neither glutathione (10 μM) nor vitamin C (250 μM) significantly affected DEPs stimulated IL-8 release (105 ± 36% and 96 ± 34% respectively, p > 0.05, n = 4). However, in acute stimulation experiments, DEP_50_ caused a transient dose-dependent increase in activation of the MAPK pathway, with peak stimulation occurring 10 minutes after DEPs exposure (Figure 1C).

### Cell viability following exposure to DEP

**Figure 2:**
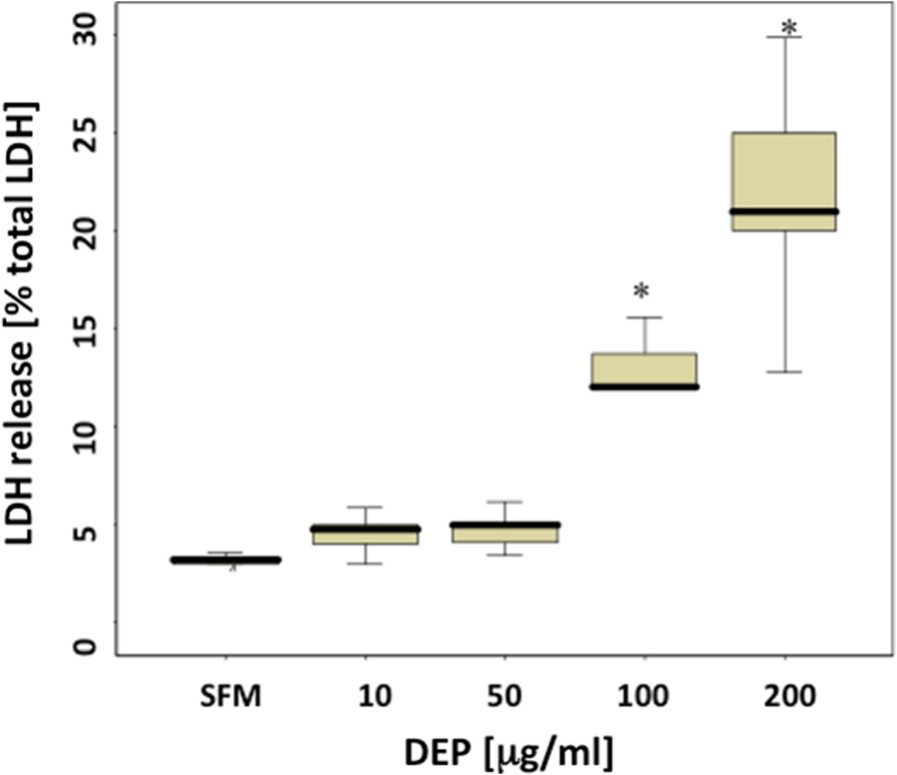
**PBEC viability following 24 h exposure to DEP.** LDH release was used as a measure of DEP-induced cytotoxicity and was expressed relative to total cellular LDH. * p < 0.001 (n = 10).

Exposure of PBEC to DEPs for 24 h elicited a dose dependent increase in LDH reaching significance at concentrations greater than 50 μg/ml (Figure 2). Carbon black did not lead to an increase in LDH release above control (SFM = 4 (3.8-4.2) *versus* CB 200 μg/ml = 4.5 (4.2-5.1) % LDH release, p > 0.05), indicating that the toxic effects of DEPs as measured by LDH release are due to the adsorbed substances on the surface of the particles, rather than the carbon core. As exposure of PBECs to doses of DEPs up to 50 μg/ml did not significantly affect cell viability, this dose was chosen for studies of the mechanisms of DEPs-induced IL-8 production.

### Involvement of EGFR

**Figure 3:**
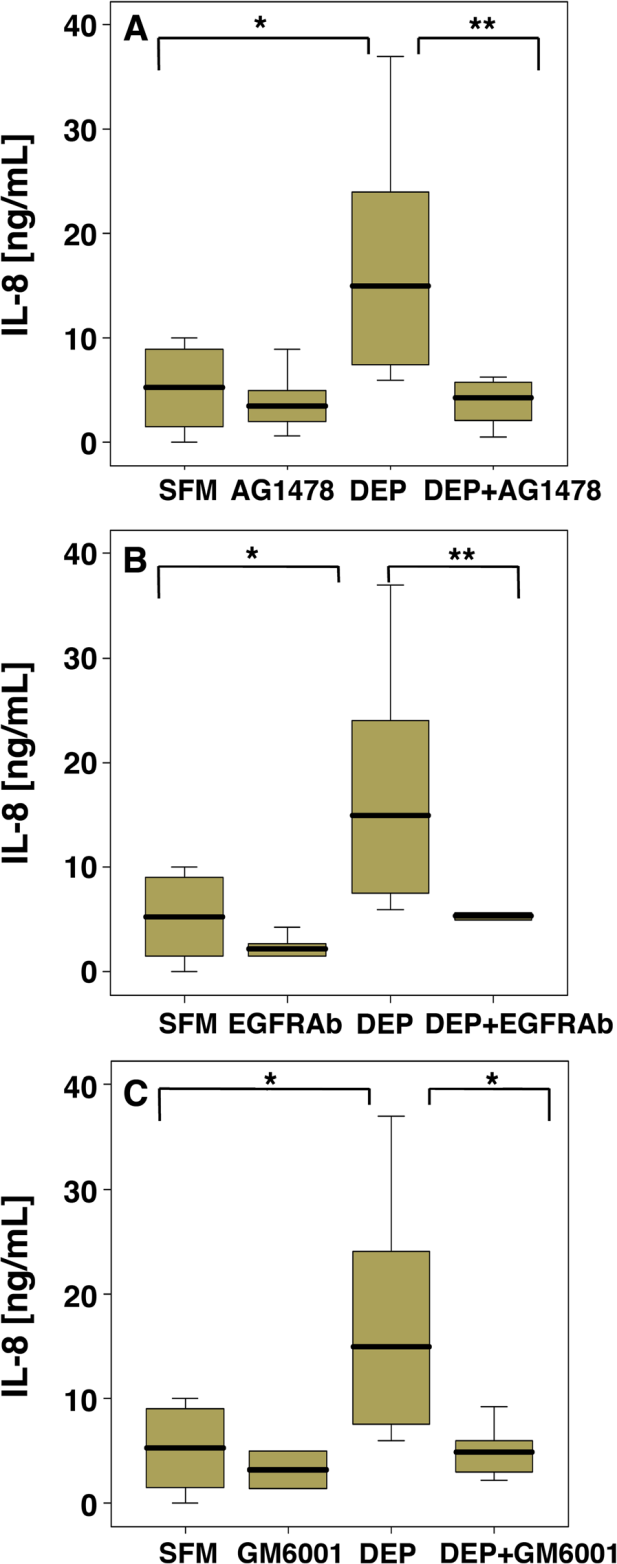
**IL-8 release from PBEC treated with DEP 50 μg/ml in the absence or presence of EGFR inhibitors.** PBEC were exposed for 24 h to DEP_50_ alone or in the presence of 1 μM AG1478 **(A)**, EGFR neutralising antibody (EGFRAb) (500 μg/ml) **(B)** or 1μM GM6001 **(C)**. * p < 0.05 ** p < 0.01 (n = 10) compared with SFM control.

Since we have previously demonstrated that secretion of IL-8 in response to cigarette smoke extract involves EGFR activation via autocrine ligand shedding [11], we next evaluated the role of the EGFR in DEP-stimulated IL-8 expression. When primary bronchial cells were exposed to DEP_50_ in the presence of an EGFR-selective tyrosine kinase inhibitor, AG1478, IL-8 release was significantly reduced (Figure 3A). Application of neutralizing antibodies for the EGFR also significantly reduced DEP_50_-induced IL-8 release (Figure 3B), indicating that activation of the EGFR had taken place through ligand binding to the receptor. As many agents activate the EGFR through release of autocrine ligands, we next investigated whether ligand shedding was associated with DEPs-induced EGFR activation. Thus, when PBEC were pre-treated with the broad metalloproteinase inhibitor (GM6001) IL-8 release was also reduced (Figure 3C) suggesting that IL-8 release was dependent on proteolytic cleavage of membrane bound EGFR agonists.

### Involvement of autocrine ligands

**Figure 4:**
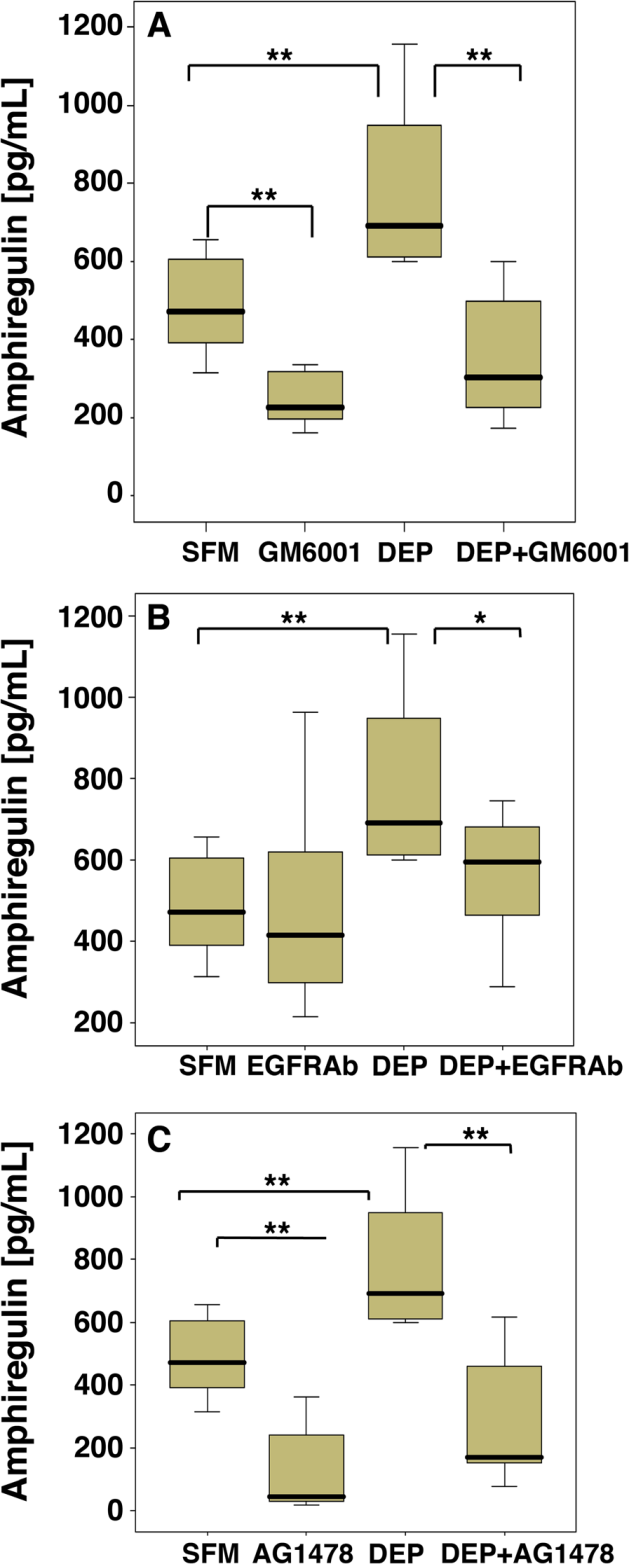
**The effect of DEP on AR protein expression in PBEC.** PBEC were exposed to DEP 50 μg/ml for 24 h in the absence or presence of GM6001 **(A)**, an EGFR neutralising antibody (EGFRAb) **(B)** and AG1478 **(C)**. AR release into the medium was measured by ELISA. p < 0.05 ** p < 0.01 n = [10] compared with SFM.

To further investigate the involvement of EGFR ligands in DEPs-stimulated IL-8 release from PBEC, we focused on three of the major EGFR ligands expressed by epithelial cells: TGFα, AR and HB-EGF. It has been demonstrated that AR plays a major role in DEPs induced GM-CSF release in a bronchial epithelial cell line [25]. Therefore we first investigated the effect of DEP_50_ on AR gene expression and release. In contrast with the previous study [25], we failed to observe significant induction of AR mRNA expression at 6 or 18 h (Table 1). However, despite a lack of mRNA induction, enhanced secretion of AR was detected in the culture medium of DEPs-exposed cells at 24 h (Figure 4) suggesting that accumulation of AR is mainly due to increased shedding rather than increased mRNA expression. In order to further investigate the relationship between AR shedding of and IL-8 release, we examined the effect of GM6001, EGFR-neutralizing antibodies and AG1478. This showed that the increase in AR release in response to DEP_50_ were reduced (Figure 4A) by GM6001 indicating that the increased levels of AR in PBEC culture supernatants is due to proteolytic shedding from the membrane. Blockade of the EGFR using a neutralizing antibody also led to a modest but statistically significant reduction of AR levels (Figure 4B). Inhibition of EGFR phosphorylation using AG1478 led to a significant reduction of AR (Figure 4C), indicating that activation of the EGFR is needed for sustained AR release.

**Table 1:** **The effect of DEP**_**50 **_**on median EGFR ligand gene expression**

	**AR median (IQR)**	**HB-EGF median (IQR)**	**TGFα median (IQR)**
SFM	2.8 (1.5-3.2)	2.0 (1.8-2.4)	2.2 (1.8–3.1)
DEP 6 h	4.1 (1.1-9.2)	4.1 (2.6-5.2)	1.5 (1.3–7.1)
DEP 18 h	2.2 (1.2-3.2)	2.0 (1.8-5.8)	4.0 (1.8–5.9)*

PBECs were exposed to DEP 50 μg/ml for 6 or 18 h and mRNA levels of the EGFR ligands determined by RT-qPCR. *p < 0.05, n = 10.

**Figure 5:**
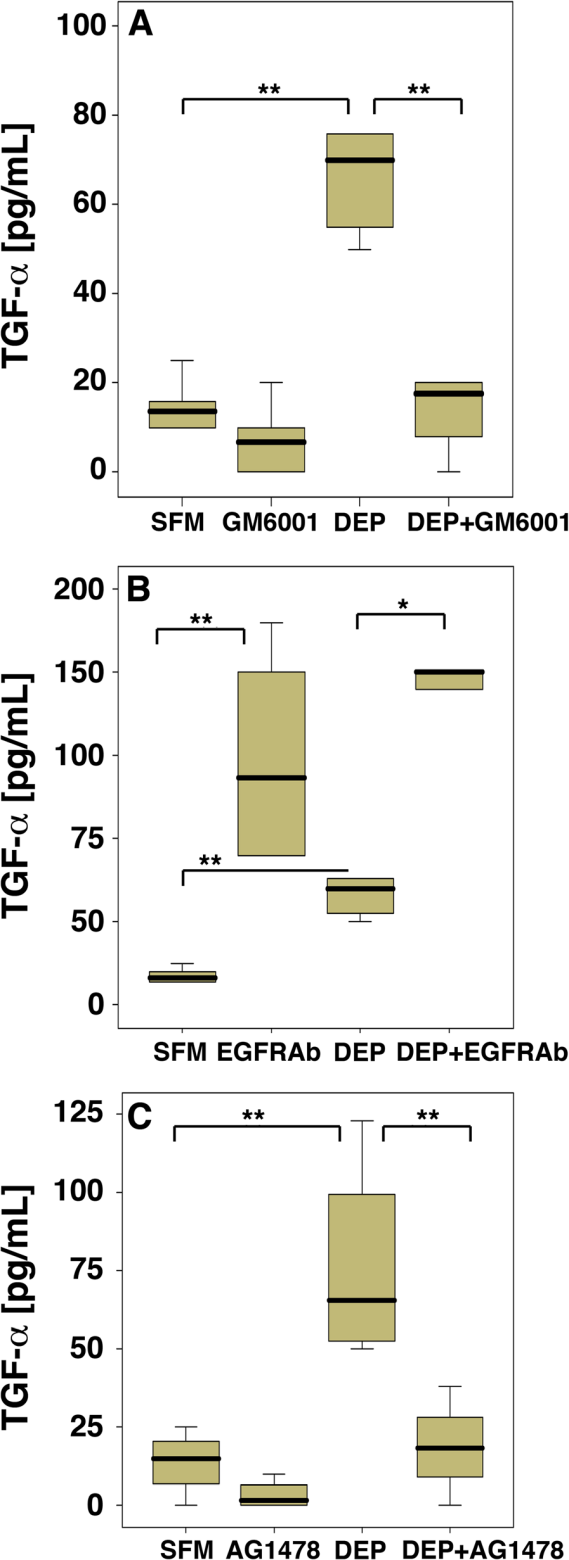
**The effect of DEP on TGF-α protein expression in PBEC.** PBEC were exposed to DEP 50 μg/ml for 24 h in the absence or presence of GM6001 **(A)**, an EGFR neutralising antibody (EGFRAb) **(B)** and AG1478 **(C)**. AR release into the medium was measured by ELISA. * P<0.01 ** P<0.005 n=(10) compared with SFM control.

As well as studying the involvement of AR in DEP-stimulated IL-8 release, we also investigated the contribution of TGF-α and HB-EGF. As for AR, levels of HB-EGF mRNA at 6 and 18 h were not significantly altered in the presence of DEP (Table 1); HB-EGF protein levels could not be measured due to a lack of suitable antibodies for ELISA. TGF-α expression was not significantly increased at 6h but did increase significantly 18 h after stimulation with DEP_50_ (Table 1). Consistent with the DEP-induced increases in mRNA there was also an increase in TGF-α release at 24 h, as measured by ELISA (Figure 5). Release of TGF-α in response to DEP_50_ was significantly reduced by GM6001 (Figure 5A), indicating that the increased TGF-α was also due to shedding of pro-TGF-α from the epithelial cell membrane. In contrast with its effect on AR accumulation, the EGFR antibody caused a significant increase in TGF-α accumulation (Figure 5B). These data suggest that the EGFR-neutralizing antibody prevents ligand binding and internalization of the receptor-bound ligand whereas in the absence of the antibody, TGF-α is rapidly utilized by the cells. Inhibition of EGFR phosphorylation using AG1478 led to a highly significant reduction in TGFα levels (Figure 5C), as was observed for AR.

**Figure 6:**
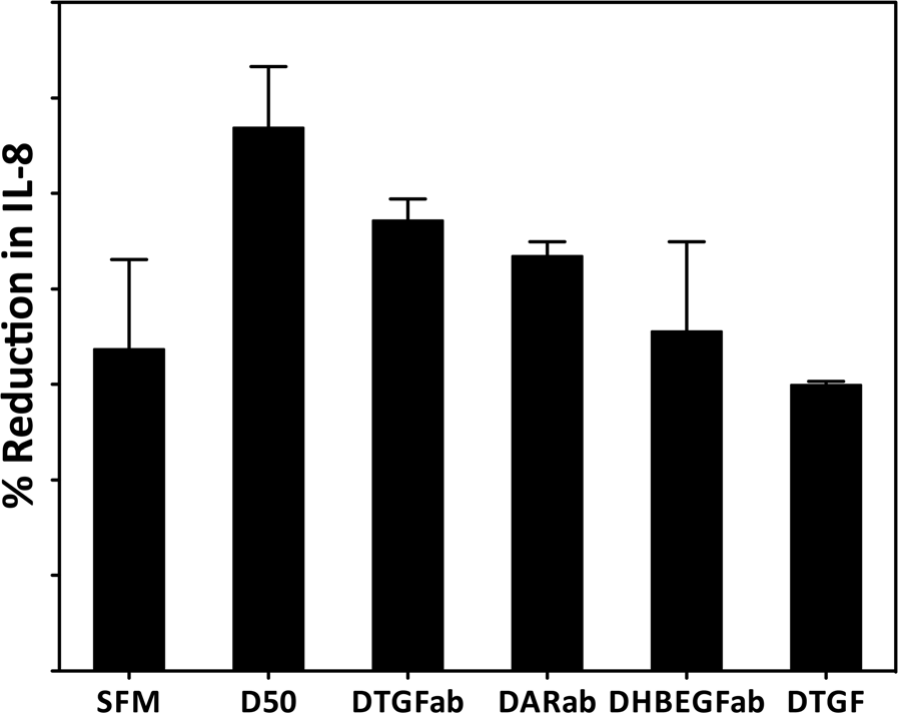
**The effect of AR, HB-EGF, and TGF-α neutralising antibodies on DEP induced IL-8 release.** The relative release of IL-8 from PBECs after exposure to DEP 50 μg/ml for 24 h in the absence or presence of TGFα, HB-EGF or AR neutralising antibodies was determined. Data show mean ± SD (n = 3). SFM = serum free medium, D50 = DEP 50 μg/ml, DTGFab = D50 + antibody to TGF-α, DARab = D50 + antibody to AR, DHBEGFab = D50 + antibody to HB-EGF, DTGF = D50 + antibody to all three ligands (TGF-α, AR, HB-EGF).

In order to determine the relative contribution of each of the three EGFR ligands to the release of IL-8, specific neutralizing antibodies were tested. This demonstrated that the release of IL-8 involved all three EGFR ligands. AR was the predominant ligand, mediating approximately 37% of the IL-8 response, whereas HB-EGF and TGF-α mediated approximately 18-24% each (Figure 6). In combination, the antibodies blocked DEP_50_-stimulated IL-8 release, consistent with the suppression of IL-8 release observed in the presence of EGFR neutralizing antibodies or the AG1478 EGFR-selective tyrosine kinase inhibitor.

## Discussion

Short term in vivo exposure to DEPs induces a marked leukocytic infiltration in the airways of healthy human volunteers involving neutrophils, lymphocytes and mast cells [5], which is associated with enhanced expression of IL-8 [19]. Although, the production of IL-8 by bronchial epithelial cells in response to DEPs is well recognized, no studies had previously explored the EGFR as a mediator of DEP-induced IL-8 release in primary human bronchial epithelial cells. Thus, our finding that DEPs induces the expression and release of the EGFR ligand TGF-α, as well as the release of AR and that neutralizing antibodies block DEP-induced IL-8 release indicates a novel, causal relationship between autocrine EGFR ligand release and IL-8 production in response to DEPs. These findings also suggest that this effect is due to activation of metalloproteinases, possibly in response to adsorbed substances, found on the surface of DEPs. Therefore the effect of DEPs on EGFR ligand processing and gene expression probably explains their ability to induce a pro-inflammatory response.

A previous study [25] demonstrated that in a human bronchial epithelial cell line (16HBE 14o-), DEPs and particulate matter <2.5 μm led to AR secretion through the activation of the EGFR and Erk (extracellular signal-regulated kinases) MAP kinase pathway by EGFR transactivation via oxidative stress. AR was also demonstrated to induce granulocyte macrophage-colony-stimulating factor (GM-CSF) release in this study, although the *in vivo* relevance of this remains to be established, as induction of GM-CSF expression following acute exposure to DEPs was not observed in a previous in vivo study [19]. In our studies using primary bronchial epithelial cells, we found many similarities with the previous *in vitro* study [25], including Erk activation, there were also notable differences. Thus, the effect of the DEPs in our study was not linked to oxidative stress. This was not surprising as the DEPs that we studied were not freshly generated and are more likely to represent ambient environmental particles rather than those inhaled when in heavy traffic. DEPs are not a single fixed particle type but their composition and hence biological activity may vary depending on many factors including the fuel source, other environmental factors including pollutants and allergens as well as time elapsed since combustion [26,27]. There may also be some differences between ‘fresh’ DEPs, which have not undergone atmospheric transformation and ‘aged’ DEPs particles such as those used in the current study, which we were unable to analyze during this study. However a study examining the impact of freshly generated DEPs on epithelial cells followed by a study examining the effect of those same particles as they age with time, would be an important area for future exploration.

Secondly, we did not find any upregulation of AR gene expression, although induction of TGFα was observed. This discrepancy may simply be due to differences in the kinetics of AR expression in PBECs compared with the cell line. Indeed, it is known that induction of EGFR ligands mRNAs show different kinetic profiles, where AR and HB-EGF gene expression are induced as an early response, followed by a later response for TGF-α [28]. At the protein level, there was augmented AR release in response to DEPs, but blockade of the EGFR with neutralizing antibodies caused accumulation of TGF-α in the culture medium, rather than AR. Although the preferential accumulation of TGFα suggested that it may be the major ligand responsible for IL-8 release, experiments using specific neutralising antibodies directed against TGFα, AR and HB-EGF indicated that they each made a contribution to the overall response to DEPs. This combinatorial effect may again reflect the ability of EGFR ligands to auto- and cross-induce their expression in a temporal fashion. Consistent with the lack of effect of antioxidants, the ability of the ligand antibodies to completely suppress DEPs-induced IL-8 release suggested that ligand independent activation of the EGFR did not contribute significantly to the responses that we observed.

Previous studies using A549 cells have suggested that a variety of environmental particles (ambient particulates and inorganic particles) can elicit IL-8 production by these cells. Use of a panel of ligands known to inhibit scavenger receptors selectively blocked responses to these particles, although the epithelial scavenger-type receptor was distinct from the heparin-insensitive acetylated-LDL receptor [29]. More recently, studies with ambient particles have suggested that normal human bronchial epithelial cells recognize coarse and fine PM through toll-like receptor 2 (TLR2) [30]. The authors suggested that degradation products of bacteria are preferentially attached to coarse pollution particles, and that bacteria themselves, dead or alive are collected as components of coarse more than fine PM. Although we collected fresh DEPs and allowed them to age, they were not collected under sterile conditions. Nonetheless, it seems unlikely that microbial contamination contributes to the responses that we observed since we found that carbon black failed to elicit IL-8 release. Thus it seems more likely that non-volatile combustion products of diesel on the surface of the DEPs were responsible for the effect.

These studies indicate a direct effect of DEPs on EGFR ligand expression in primary bronchial epithelial cells. EGFR ligands are involved in a number of effects that could also potentially lead to the effects seen in chronic asthma. TGF-α is associated with mucin hypersecretion and pulmonary fibrosis as well as branching morphogenesis during lung development [31-34]. Amphiregulin (AR) is an epidermal growth factor (EGF)-related peptide that can bind to heparin and operates exclusively through the EGFR. Studies have shown that this peptide is expressed in PBEC [13,35]. Increased levels of this growth factor are associated with malignancy in the breast [36]. HB-EGF, another EGFR ligand, has been shown to mediate mucin transcription [37] and fibroblast proliferation [38]. This growth factor has been shown to be upregulated by vanadium, through an oxidant dependent mechanism, as well as cigarette smoke extract [39]. One of the limitations in our study is that we did not fully explore the dynamic relationship between the antibodies utilized and the ligands and receptors being explored. Future work should explore the specificity, dose response relationship as well as the kinetics of each of the antibodies utilized in this study to better elucidate the underlying mechanisms as well as specificity of each of the antibodies. Further exploration should also be focused on studying the effect of knock down of specific ligands.

In conclusion, the epithelial IL-8 response to DEPs occurs through the EGFR via shedding and involvement of multiple autocrine EGFR ligands. The ability of DEPs to induce expression and release of ligands for the EGFR suggests that they may have other direct effects on epithelial function such as induction of proliferation or differentiation linked to mucus production.

## Abbreviations

DEP: Diesel exhaust particles; EGFR: Epithelial growth factor receptor; IL-8: Interleukin-8; AR: Amphiregulin; TGFα: Transforming growth factor alpha; HB-EGF: Heparin-binding EGF-like growth factor.

## Competing interest

The authors declare that they have no conflict of interest relevant to this manuscript.

## Authors’ contributions

SP: Conducted all experiments except western blots, data analysis, write up manuscript. LMH: Conducted the western blot experiments and their data analysis and its incorporation in the manuscript. SMP: Conducted cultures of primary bronchial epithelial cells. STH: Evaluation of the data, critical review of data and the manuscript. Conception of the studies. AJF: Evaluation of the data, critical review of data and the manuscript. Conception of the studies. DED: Evaluation of the data, critical review of data and the manuscript. Conception of the studies. All authors read and approved the final manuscript.
